# Gut microbiota-mediated regulation of lipid metabolism by single herbal medicines: a review focused on cold/hot properties

**DOI:** 10.1186/s13020-025-01301-z

**Published:** 2026-01-13

**Authors:** Jiale Fang, Siwen Wang, Aijun Quan, Xinyu Zhu, Baitao Li, Deyou Jiang

**Affiliations:** 1 The Third Affiliated Hospital, Heilongjiang University of Chinese Medicine, No. 2 Xiangjiang Road, Xiangfang District, Harbin, China; 2 The Second Affiliated Hospital, Heilongjiang University of Chinese Medicine, No. 411, Gogol Street, Nangang District, Harbin, China; 3 Heilongjiang University of Chinese Medicine, No. 26, Heping Road, Xiangfang District, Harbin, China

**Keywords:** Herbal medicine, Cold/hot properties, Gut microbiota, Lipid metabolism, Personalized treatment

## Abstract

Single herbal medicines (SHMs) possess distinct advantages in regulating lipid metabolism, particularly through modulating the composition and function of the gut microbiota. Compared with herbal formulas, SHMs are characterized by traceable active components, clear pharmacological targets, and greater reproducibility. However, their intrinsic cold/hot properties must not be overlooked, as inappropriate or prolonged use may disturb the body’s holistic balance and pose health risks. Therefore, it is essential to systematically classify lipid-lowering SHMs according to their cold/hot properties and establish a “medicinal properties–microbiota” framework to guide personalized treatment strategies for hyperlipidemia. We searched PubMed, Web of Science, EMBASE, the Cochrane Library, the Chinese Biomedical Literature Database, and CNKI for studies published up to August 2025. Studies reporting lipid-lowering effects of SHMs through gut microbiota regulation were screened and analyzed. Fifty-seven SHMs were included, comprising 24 cold/cool-property, 20 warm/heat-property, and 13 neutral-property medicines. These SHMs improved lipid metabolism mainly by modulating the gut microbial composition and metabolites such as short-chain fatty acids (SCFAs), lipopolysaccharides (LPS), bile acids (BAs), and trimethylamine N-oxide (TMAO). Shared microbiota changes included a reduced Firmicutes/Bacteroidetes ratio and increased abundance of Akkermansia, Lactobacillus, Ruminococcaceae, and Clostridium. A new pattern has been identified: cold/cool-property SHMs mainly targeted environmentally adaptive microbial taxa, warm/heat-property SHMs enriched *Akkermansia muciniphila* and promoted SCFA production, whereas neutral-property SHMs exerted broad-spectrum regulation to maintain microbial homeostasis. In summary, SHMs regulate lipid metabolism through multiple microbiota-related pathways, and the effects are closely associated with their cold/hot properties. The classification of SHMs according to the four natures provides a theoretical basis for personalized treatment and may guide the safe and effective use of SHMs for the treatment of dyslipidemia.

**Graphical abstract:**

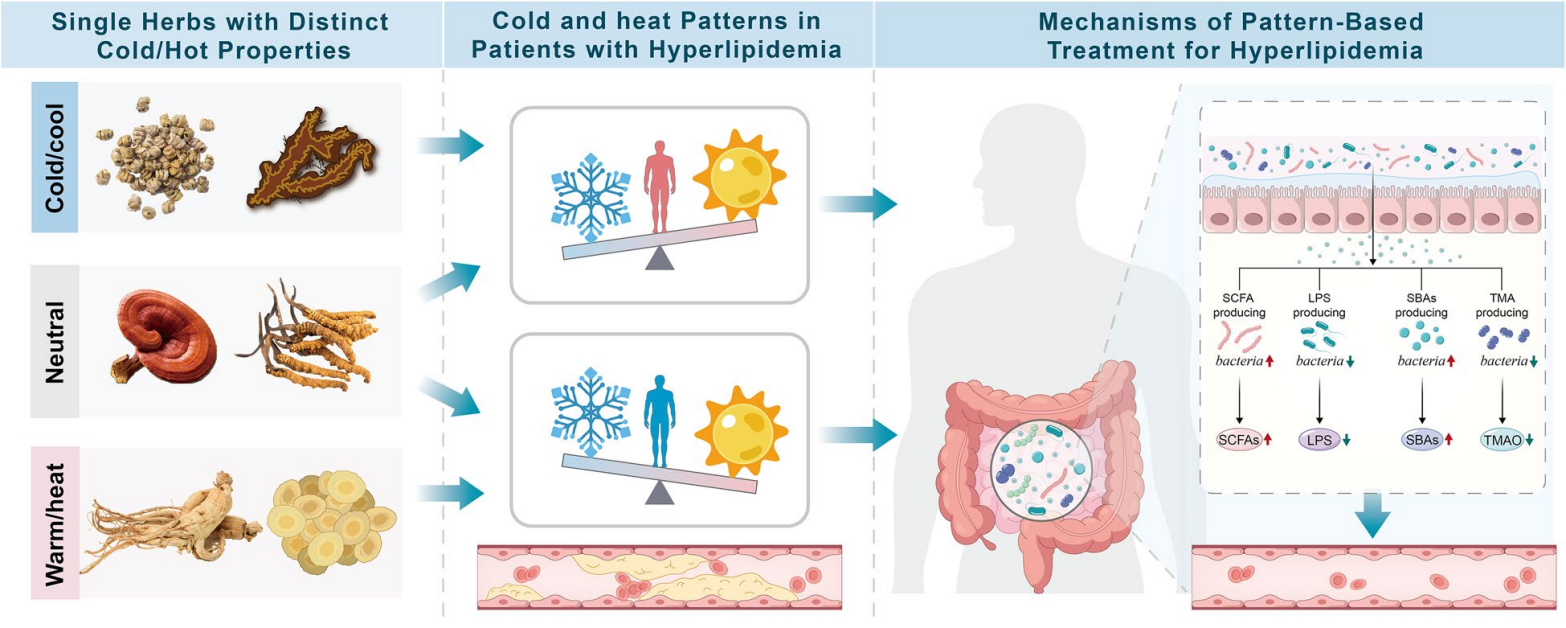

## Introduction

Lipid metabolism involves a sophisticated network of biochemical processes regulated by lifestyle habits, nutritional status, genetic factors, and insulin resistance. Dysregulation of lipid metabolism is primarily reflected in elevated serum levels of total cholesterol (TC) and/or triglycerides (TGs) and broadly encompasses various forms of dyslipidemia, including low levels of high-density lipoprotein cholesterol (HDL-C) [1]. These abnormalities significantly contribute to the pathogenesis of atherosclerotic cardiovascular disease [2] and are closely linked to conditions such as obesity [3], nonalcoholic fatty liver disease (NAFLD) [4], diabetes mellitus [5], neurodegenerative disorders [6], and even cancer [7–9]. Although current first-line lipid-lowering agents are effective, they may lead to undesirable side effects in certain patients, including hepatic dysfunction, muscle injury, and hyperglycemia [10]. Consequently, the search for novel lipid-lowering strategies that are both safe and efficacious remains a pressing clinical priority.

In recent years, many animal and clinical studies have highlighted the pivotal role of the gut microbiota and its metabolites in the regulation of lipid metabolism [11–15]^.^ The underlying mechanisms involve direct or indirect interactions with distal organs, as well as modulation via neural signaling pathways and gut-derived hormones [16]. Accordingly, targeting the gut microbiota and its metabolic products has emerged as a promising strategy for managing dyslipidemia. Nonetheless, the clinical application of this approach remains constrained by the lack of microbiota-targeted agents with well-defined efficacy.

Herbal medicine (HM), derived from natural sources such as plants, animals, and minerals—primarily plant-based—has received increasing research attention. Accumulating evidence indicates that HMs can influence lipid metabolism by modulating the gut microbiota and its metabolites [17, 18]. Among these, single HMs (SHMs) has gained prominence because of their traceable active components, specific targets, favorable absorption, and low toxicity profile [19–22]. However, unlike multiherb formulas, SHM may disrupt the body’s holistic balance, potentially exacerbating yin-yang or cold–heat imbalances—concepts that correspond to subhealth conditions in Western medicine—thereby increasing the risk of disease. Despite numerous reports supporting the lipid-regulating effects of SHM via gut microbiota modulation, clinical guidance remains limited, and comprehensive assessments of potential adverse effects are still lacking.

This review systematically surveyed literature from the past ten years using PubMed, Web of Science, EMBASE, Cochrane Library, the Chinese Biomedical Literature Database, and CNKI, with keywords including herbal medicine, herbs, gut microbiota, gut–liver axis, lipid metabolism, obesity, hyperlipidemia, and metabolic syndrome. It first summarizes the principal mechanisms through which SHMs regulate lipid metabolism by modulating the gut microbiota and its metabolites. It then explores the classification, therapeutic indications, and potential clinical risks of SHM on the basis of the traditional Chinese medicine (TCM) theory of the “Four qi” (Chinese romanization: Si Qi, including cold, hot, warmth, and coolness) and proposes rational strategies for clinical use. Finally, on the basis of this classification framework, we systematically review recent advances in the use of cold/cool-, warm/heat-, and neutral-property SHM for lipid regulation. Overall, this review provides a novel perspective that integrates traditional TCM theory with modern microbial ecology and offers a theoretical basis for future research and clinical application in the treatment of hyperlipidemia.

## Mechanisms of lipid metabolism regulation by HM via the gut microbiota

**Fig. 1:**
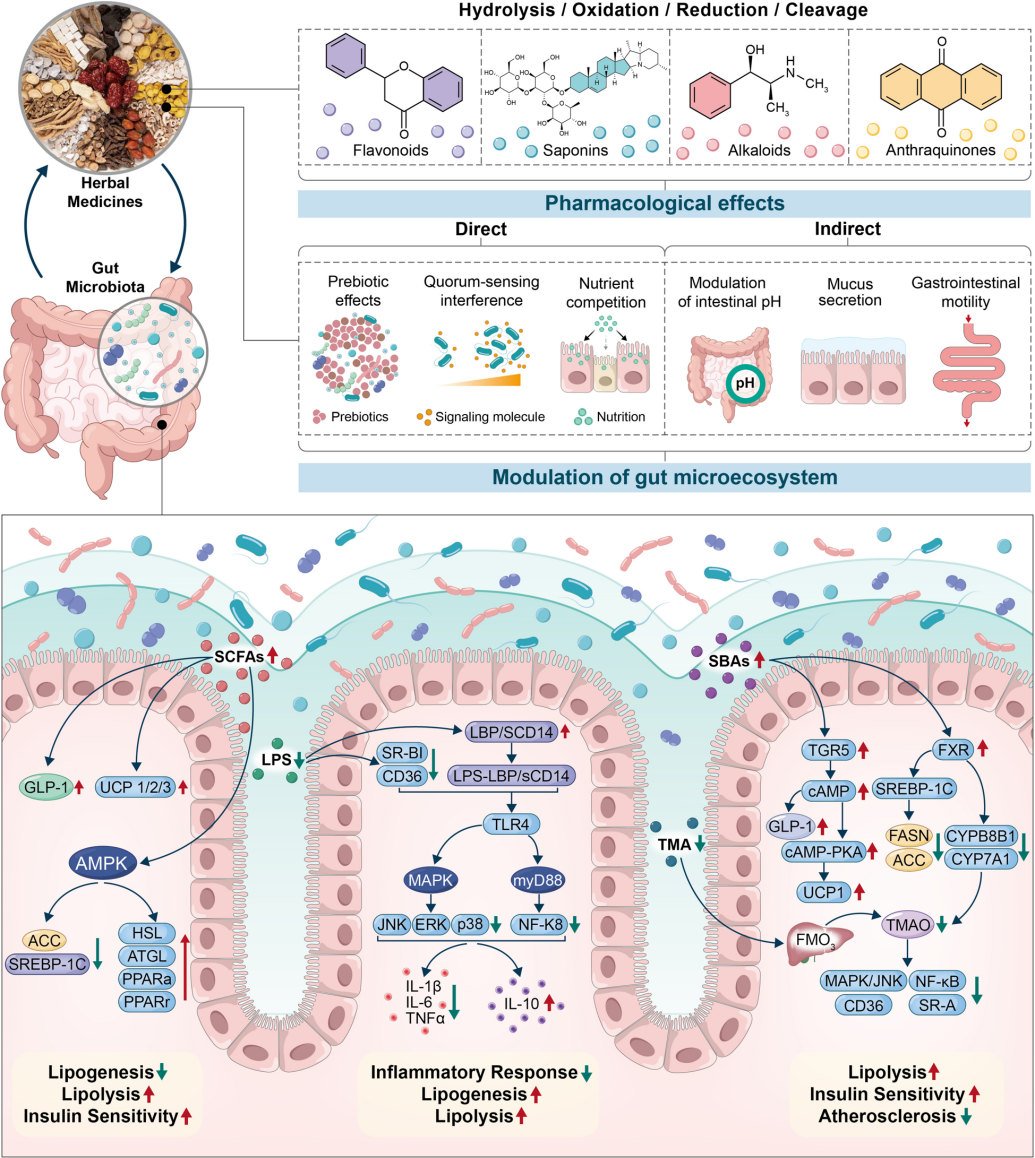
Mechanisms by which HMs interact with the gut microbiota to improve lipid metabolism

The gut microbiota is a fundamental component of the human digestive system [11, 23, 24]. Emerging evidence indicates that the dynamic interplay between HM and the gut microbiota is crucial in the regulation of lipid metabolism [25–28]. As illustrated in Fig. 1, through a series of biotransformation processes—such as hydrolysis, oxidation, reduction, and cleavage—gut microbes convert the chemical constituents of HM into metabolites with reduced polarity, increased lipophilicity, and enhanced bioactivity, making them more readily absorbed and pharmacologically potent within the host [29, 30]. In parallel, HMs modulate the gut microecosystem through both direct mechanisms (e.g., restructuring the microbial composition) and indirect mechanisms (e.g., altering the intestinal pH, gastrointestinal motility, and metabolic milieu). These changes impact the production of key microbial metabolites, including short-chain fatty acids (SCFAs), bile acids (BAs), trimethylamine-N-oxide (TMAO), and lipopolysaccharide (LPS) [25, 31–33]. These metabolites exert systemic and localized effects on various host organs and tissues—such as the liver, adipose and muscle tissues, and the brain—where they participate in inflammation regulation, maintenance of intestinal barrier integrity, and modulation of fatty acid metabolism, thereby influencing overall lipid homeostasis.

SCFAs, including acetate, propionate, and butyrate, are crucial microbial metabolites generated via fermentation of indigestible dietary fibers by the gut microbiota [34]. SCFAs regulate lipid metabolism through both direct and indirect mechanisms. They can directly activate the AMP-activated protein kinase (AMPK) pathway by increasing the intracellular AMP/ATP ratio or indirectly modulate AMPK signaling via the engagement of the G protein-coupled receptors GPR41 and GPR43, thereby increasing fatty acid oxidation [35, 36]. Upon AMPK activation, the activities of hormone-sensitive lipase (HSL) and adipose triglyceride lipase (ATGL) are increased, whereas hepatic nuclear receptors such as peroxisome proliferator-activated receptor alpha (PPARα) and gamma (PPARγ) are modulated to promote β-oxidation and lipolysis [37, 38]. In addition, SCFAs suppress lipogenic gene expression—including sterol regulatory element-binding protein-1 (SREBP-1) and acetyl-CoA carboxylase—thereby reducing fatty acid synthesis [39, 40]. SCFAs also modulate the composition of the gut microbiota to influence the secretion of gut hormones such as glucagon-like peptide-1 (GLP-1), ultimately improving insulin sensitivity and regulating adipose tissue storage [41]. Notably, emerging evidence suggests that SCFAs, especially propionate, can influence intestinal cholesterol absorption via the Treg/IL-10/Niemann‒Pick C1-like 1 signaling axis and enhance macrophage polarization in adipose tissue, collectively contributing to a more favorable lipid metabolic environment [42].

Certain facultative and anaerobic bacteria in the gut are capable of metabolizing BAs into secondary BAs [43–45]. These secondary metabolites primarily regulate lipid and glucose metabolism by activating two key receptors: farnesoid X receptor (FXR) and Takeda G protein-coupled receptor (TGR5) [46]. In the liver, FXR activation suppresses the expression of sterol regulatory element-binding protein-1c (SREBP-1c) and its downstream lipogenic enzymes, including fatty acid synthase (FASN) and acetyl-CoA carboxylase, thereby inhibiting triglyceride synthesis. Concurrently, FXR downregulates cholesterol 7α-hydroxylase (CYP7A1) and cytochrome P450 family 8 subfamily B member 1, reducing the conversion of cholesterol into BAs and maintaining the cholesterol–BA metabolic balance [47, 48]. In addition, TGR5 is expressed predominantly in the intestine, brown adipose tissue, and skeletal muscle [49–51]. Upon activation by BAs, TGR5 stimulates uncoupling protein 1 (*Ucp1*) expression via the cyclic AMP (cAMP)/protein kinase A (PKA) signaling pathway in adipose tissue, thereby promoting thermogenesis and reducing lipid accumulation. In intestinal L-cells, TGR5 activation increases cAMP levels, enhances GLP-1 secretion, improves insulin sensitivity, and facilitates glucose homeostasis [46].

TMAO is generated in the liver via the enzymatic activity of flavin-containing monooxygenase 3 (FMO3), which oxidizes trimethylamine (TMA)—a compound derived from gut microbial fermentation of phosphatidylcholine, choline, and L-carnitine [52]. Elevated circulating TMAO levels have been shown to activate an FXR-dependent negative feedback mechanism that inhibits hepatic expression of CYP7A1, thereby reducing the conversion of cholesterol into BAs and promoting its deposition within vascular walls [53]. Moreover, TMAO enhances atherosclerosis progression by activating the mitogen-activated protein kinase (MAPK)/c-Jun N-terminal kinase (JNK) and NF-κB signaling pathways, leading to the upregulation of scavenger receptors such as cluster of differentiation 36 and scavenger receptor class A. This phenomenon promotes the uptake of ox-LDL by macrophages, facilitates foam cell formation, and accelerates vascular inflammation and plaque development [52, 54, 55].

LPS, also known as endotoxin, is a structural component of the outer membrane of gram-negative bacteria in the intestinal tract [56, 57]. Chronic exposure to a high-fat diet, obesity, and elevated levels of free fatty acids (FFAs) can lead to gut dysbiosis, increased LPS production, and compromised intestinal barrier function [58]. Circulating LPS promotes the expression of scavenger receptors such as scavenger receptor class B type I and cluster of differentiation 36, enhancing the uptake of low-density lipoprotein (LDL), oxidized LDL (oxLDL), and fatty acids by macrophages and adipocytes [59]. Additionally, LPS forms a complex with LPS-binding protein and soluble Cluster of Differentiation 14, which activates the MAPK signaling cascade (including ERK, p38, and JNK) via Toll-like receptor 4 (TLR4) and further initiates the NF-κB pathway through the TLR4/MyD88 axis, leading to increased expression of inflammatory cytokines such as TNF-α, IL-1β, IL-6, and IL-10 [56, 60]. These proinflammatory mediators impair insulin signaling, induce insulin resistance, and disrupt normal adipose tissue function [61, 62].

The interplay between HM and the gut microbiota is multifaceted, dynamic, and highly individualized [63]. The lipid-lowering efficacy of HM is not universally beneficial or detrimental, as it depends on the plasticity of the gut microbiota and the individual’s TCM syndrome type [64–66]. Recent studies have demonstrated that different TCM syndromes correspond to distinct microbial profiles in the gut [67, 68]. Therefore, under the guidance of TCM syndrome differentiation, HMs can be applied in a targeted manner to modulate specific microbial populations, thereby enhancing their lipid-lowering effects and effectively correcting gut microbiota-related dyslipidemia.

## Classification of SHM properties and corresponding clinical indications

TCM, grounded in ancient Chinese philosophical principles, emphasizes the maintenance of internal physiological balance—such as between qi and blood, cold and heat, deficiency and excess, and dryness and dampness—as the foundation for health and adaptability to environmental challenges [69]. One of the primary aims of SHM is to restore this internal balance when disrupted. Within the TCM framework, the equilibrium between cold and heat is particularly critical. On the basis of their influence on this balance, SHMs are traditionally classified into four properties: cold, hot, warmth, and coolness [70], with the cold/coolness and warmth/hot properties being mutually opposing. In addition, SHMs with neutral-property (Chinese romanization: ping) are also widely used for their gentle effects. In order to simplify their practical use, SHMs are commonly grouped into three categories: cold/cool-, warm/heat-, and neutral-property [71, 72].

**Fig. 2:**
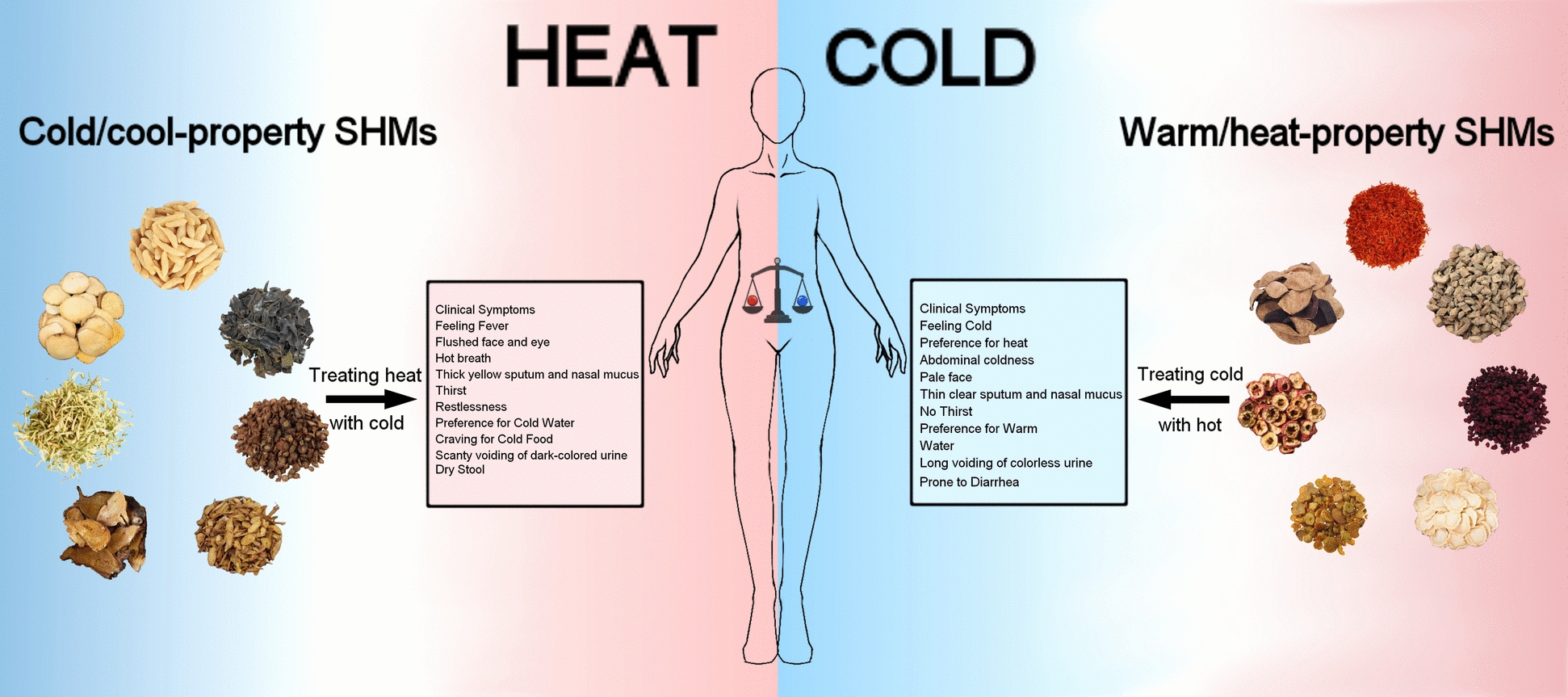
Syndrome-guided application of cold/cool- and warm/heat-property HMs for the management of lipid metabolic disorders (The color differences between the two sides of the human body represent distinct body constitutions: red indicates heat syndrome and blue indicates cold syndrome, corresponding to the use of cold/cool- and warm/heat-property HMs, respectively)

In the treatment based on pattern identification, the medicinal properties of an SHM determine its suitability for specific pathological states (Fig. 2). Cold/cool-property SHMs are used to treat heat syndrome (e.g. fire hyperactivity) and have properties such as heat-clearing, toxinremoving, cool-blood, and resolve swelling effects. They are commonly prescribed for symptoms such as fever, restlessness, thirst, preference for cold drinks, and hard stools. SHMs with warm/heat-property are used to warm the body, dispel pathogenic cold, improve circulation, and increase body temperature. These are appropriate for cold syndromes, which typically present as cold intolerance, lack of thirst, preference for warm drinks, and loose stools [73]. Neutral-property SHMs are suitable for individuals with balanced constitutions or when cold–heat tendencies are not clearly defined. They are frequently used as adjuncts in managing concurrent symptoms.

In summary, the cold/hot property classification system is a cornerstone of treatment based on pattern identification in TCM. This categorization is grounded in the differing physiological responses and therapeutic outcomes elicited by each SHM [74]. SHMs with cold/cool-, warm/heat-, or neutral-property exert effects by modulating the body’s cold‒heat balance and restoring systemic function; therefore, their clinical application should be aligned with the patient’s body constitution [75]. Similarly, when using SHMs to treat dyslipidemia, it is essential to evaluate the patient’s cold–heat syndrome through TCM diagnostics and select herbal medicines accordingly. Failure to do so may lead to mismatches between the medicinal cold/hot property and the patient’s body constitution, resulting in reduced efficacy or even adverse effects that could aggravate the disorder [76–78].

## Regulation of lipid metabolism by SHM via the gut microbiota

### SHM with cold/cool-property

Cold**/**cool -property SHMs possess heat-clearing, toxinremoving, cool-blood, and resolve swelling, making them suitable for treating heat-related syndromes. Our analysis revealed 24 SHMs with the ability to regulate lipid metabolism through the modulation of the gut microbiota (Table 1). Notable examples include *Pueraria lobata* starch [79], *mulberry leaf* water extracts (MLEs) [80], *rotundic acid* [81], *berberine* (BBR) [82], *Plantago asiatica L.* polysaccharide [83], *Ophiopogon* polysaccharide (MDG-1) [84], 95% ethanol extract of *mulberry fruit* [85], *and andrographolide* [86], as well as polysaccharides from *Apocynum venetum* leaves [87] and *Polygonatum odoratum* polysaccharides [88], all of which have been reported to increase the production of SCFAs. For example, Zhao et al. [80] demonstrated that MLEs increased the relative abundance of Bacteroidetes while reducing the Firmicutes-to-Bacteroidetes (F/B) ratio, upregulated *Ruminococcaceae* and *Lactobacillaceae*, and suppressed *Erysipelotrichaceae* levels, collectively leading to elevated butyrate and acetate concentrations. Similarly, MDG-1 significantly increased acetate, valerate, propionate, and butyrate levels by reshaping the gut microbial composition and subsequently activated hepatic GPR41 and GPR43 receptors, along with the AMPK–SREBP-1c signaling pathway [84]. Su et al. [86] demonstrated that *andrographolide* not only elevates SCFA levels but also enhances the expression of *Occludin* and *ZO-1* (tight junction proteins), thereby preserving intestinal barrier integrity. This action reduces the leakage of LPS into the bloodstream and contributes to its anti-inflammatory and antioxidant effects.

**Table 1:** Summary of studies on cold/cool-property SHMs regulating lipid metabolism via the gut microbiota

SHM	Extract	Experimental subject	Improvement index	Changes in gut microbiota composition	Mechanisms
*Salvia miltiorrhiza*	Sal75	HFDF SD rats	Body weight,BFI, Waistline, GLUCOSE, TG, TC, LDL-C, HDL-C, FFAs, Histopathological changes in liver	*Facklamia*, *Corynebacterium*, *Psychrobacter*, *Quinella*, *Blautia*	1. *Sal* increased SCFA-producing gut microbiota and promoted lipolysis through the cAMP/PKA/HSL signaling pathway 2. *Sal* modulates bile acid metabolism. [89].
*Laminaria japonica*	LJP61A	HFDF C57BL/6 mice	Body weight, Liver weight, Epididymal fat weight, Epididymal fat cell size, Hepatocyte lipid deposition, FBG, Fasting insulin, HOMA-IR, Liver histopathology, Levels of inflammatory cytokines	ratio of Firmicutes/Bacteroidetes, *Akkermansia*	1. LJP61A treatment significantly decreased serum level of LPS 2. LJP61A enhances intestinal barrier integrity. [104].
*Pueraria lobata (Willd.) Ohwi*	PLS	HFDF C57BL/6 J mice	Body weight, Serum IL-6, Serum TNF-α, Liver histology score, ALT, AST, TC, TG, LDL-C	*Lactobacillus*, *Bifidobacterium* and *Turicibacter*, *Desulfovibrio*	PLS increased the levels of SCFAs in feces. [79].
*Alisma orientale (Sam.) Juzep*	AOE	HFDF SD rats	Body weight, FBG, Liver histopathology, ALT, AST, TC, TG, LDL-C, HDL-C	Lachnospiraceae, Lactobacillaceae/*Lactobacillus*, Ruminococcaceae/*Ruminococcus*	1. AOE upregulated Insig1 expression, thereby inhibiting cholesterol synthesis 2. AOE treatment significantly enriched SCFAs producing bacteria. [90].
*Radix Scutellariae*	WESB	HFDF SD rats	Body weight, FBG, Fasting insulin, HOMA-IR, OGTT, IPITT, TC, TG, LDL-C	*Lactobacillus*, *Faecalibaculum*, *Blautia*, *Lachnoclostridium*, *Ruminiclostridium*_5, *Turicibacter*	WESB increased BAs production and upregulated hepatic CYP7A1 protein expression. [105].
*Mulberry leaf*	MLEs	HFDF SD rats	Weight gain rate, WAT content, BAT content, Lee’s index, TG, LDL-C, HDL-C, NEFA	ratio of Firmicutes/Bacteroidetes, *Lactobacillus*, *Ruminococcus*	1. MLEs increased the levels of SCFAs in feces 2. MLEs promoted lipid metabolism by upregulating key lipid metabolism-related genes, Pla2g2a and Plac8. [80].
*Polygonum cuspidatum*	PCPs-I	HFDF STZCC57BL/6 J mice	FBG, ITT, TG, TC, LDL-C, HDL-C, FFA, AST, ALT, FFA	*Corynebacterium*, *Ligilactobacillus*, *Oligella*, *Mammaliicoccus*, Firmicutes, Bacilli	PCPs-I treatment significantly enriched SCFA producing bacteria. [91].
Ilicis Rotundae Cortex	Rotundic acid	HFDF SD rats	Body weight, Liver index, Fat index, TG, TC, LDL-C, HDL-C, AST, ALT, Hepatic steatosis	ratio of Firmicutes/Bacteroid, *Bacteroides*, *Alloprevotella*, *Desulfovibrio*, Prevotella_9, *Lactobacillus*	Rotundic acid increased the levels of SCFAs in feces. [81].
*Coptis chinensis* Franch	BBR	HFDF C57BL/6 J mice	TG, TC, LDL-C, Liver TC, TG	Clostridium cluster XI, *Anaerostipes*, *Blautia*, *Akkermansia*, *Coprobacillus*, *Alistipes*, *Helicobacter*, *Enterorhabdus*, *Desulfovibrio*	1. BBR increased the levels of SCFAs in feces 2. BBR upregulates LDLR expression and enhances LDL uptake by HepG2 cells. [82].
*Gynostemma pentaphyllum* (Thunb.) Makino	Agl	HFDF SD rats	TG, TC, LDL-C, HDL-C, Hepatic lipid accumulation, Expression of lipid metabolism-related genes	*Lactobacillus*, *Akkermansia*, *Blautia*, Chlamydiaceae, *Lactobacillus murinus*, *Firmicutes* bacterium CAG:424, *Allobaculum stercoricanis*	1. Agl downregulates the expression of lipogenesis-related genes 2. Agl may regulate cholesterol and bile acid metabolism via the FXR-FGF15 axis. [93].
*Plantago asiatica L*	PLP	HFDF + STZ Inbred Wistar rats	Body Weight, FBG, Insulin Level, GSP, QUICKI, Scr, UA, BUN, TG, TC, LDL-C, HDL-C	*Bacteroides vulgatus*, *Lactobacillus fermentum*, *Prevotella loescheii*, *Alistipes obesi*, *Clostridium bolteae*	1. PLP increased the levels of SCFAs in feces 2. PLP suppressed oxidative stress by reducing MDA and enhancing SOD, CAT, T-AOC. [83].
*Lonicera japonica* Thunb	FFL	HFDF SD rats, RAW 264.7, HCT-116	Body Weight,Adipose Tissue Weight (Abdominal, Epididymal), Hepatic Lipid Deposition, TG, HDL-C, TG, AST, Inflammatory Markers, Tight Junction Protein Gene Expression (ZO-1, Claudin-1)	*Akkermansia* spp., ratio of Firmicutes/Bacteroidetes	1. FFL upregulated ZO-1 and Claudin-1 expression, and reduced serum PS 2. FFL inhibited LPS-induced TNF-α, IL-6, and COX-2 expression in macrophages. [106].
*Ophiopogon japonicus*	MDG-1	HFDF C57BL/6 J mice	Body weight, TG, TC, ALT, AST, Liver lipid accumulation, Inflammatory cytokines	ratio of Firmicutes/Bacteroidetes, *Alistipes*, *Ruminiclostridium*, *Rikenella*, *Butyricimonas*, *Roseburia*	1. MDG-1 increased the levels of SCFAs in feces 2. MDG-1 downregulated IL-1β, IL-4; upregulated IL-10. [84].
*Mulberry* Fruit	MFE95	HFDF ICR mice	Body Weight, Hepatic Steatosis, TC, LDL-c, Oxidative Stress Markers	*Adlercreutzia*, *Limosilactobacillus*, Clostridia_unclassified, Clostridium_XIVb, Ruminococcaceae	1. MFE95 enhanced hepatic antioxidant enzyme activities (e.g., SOD, GSH, CAT) 2. MFE95 enriched butyrate-producing bacteria. [85].
*Andrographis paniculata* (Burm. f.) Nees	Andrographolide	*db/db* mice	TC, TG, ALT, AST, FFAs, GTT, ITT, superoxide radicals, Inflammatory cytokines, HOMA-IR	ratio of Firmicutes/Bacteroidetes, *Akkermansia muciniphila*, Rikenellaceae, Odoribacteraceae, *Parabacteroides*, *Oscillospira*	1. Andrographolideincreased the levels of SCFAs in feces 2. Enhanced tight junction protein expression; reduced serum LPS 3. Anti-inflammatory and antioxidant effects(TNF-α, IL-6, ROS, GSH). [86].
*Dendrobium huoshanense*	DH	HFDF C57BL/6 J mice	Body weight, TC, TG, ALT, AST, Liver TC, TG, Hepatic steatosis score	*Bifidobacterium*, *Allobaculum*, *Helicobacter hepaticus*, *Rothia mucilaginosa*, *Clostridium celatum*	DH promoted fecal excretion of deoxycholic acid and downregulated amino acid metabolism. [95].
*Rheum palmatum L*	RHUB	HFHS C57BL/6 J mice	Body weight, fat mass, insulin, OGTT, FBG, Liver inflammation, Inflammatory cytokines, Liver TC, TG	*Akkermansia muciniphila*, *Parabacteroides*, *Erysipelatoclostridium*, *Ruminococcus* and *Peptococcus*	1. RHUB enhances gut immune barrier function by modulating the expression of antimicrobial peptide genes 2. RHUB increases Ucp1 gene expression and promotes energy metabolism 3. RHUB exerts anti-inflammatory effects (TNF-α, IL-6, IFN-γ, RANTES). [92].
*Cichorium intybus L.* (chicory)	CA	HFDF C57BL/6 J mice	Body weight, TC, TG, LDL-C, HDL-C, ALT, AST, Inflammatory cytokines, WAT weight	ratio of Firmicutes/Bacteroidetes, *Lactobacillus*, *Bacteroides*, *Alloprevotella*, Ruminococcaceae	CA exerts antioxidant, anti-inflammatory, and lipid-lowering effects via activation of the AMPK/Nrf2/NFκB pathway. [107].
*Apocynum venetum* leaves	AE	HFDF + STZ C57BL/6 J mice	TG, LDL-C, NEFA, FBG, HOMA-IR/HOMA-β, GSP, Oxidative Stress Markers	*Odoribacter*, *Anaeroplasma*, *Parasutterella*, *Muribaculum*, *Enterococcus*, *Klebsiella*, *Aerococcus*	AE increased the levels of SCFAs in feces. [87].
Bear bile powder	BBBP	HFDF C57BL/6 J mice	TG, TC, LDL-C, HDL-C, FBG, ALT, AST, TBA, Hepatic lipid accumulation, Liver histopathology	Firmicutes, Actinobacteria, Bacteroidota, *Lactobacillus*, *Bacteroides*, *Desulfovibrio*	BBBP increased serum TBA levels and activated bile acid receptors (FXR, PXR, S1pr2). [108].
*Polygonatum odoratum* (Mill.) Druce	POP	HFDF SD rats	Body weight, Epididymal and perirenal fat, Epididymal adipocyte size, Liver TC, TG, Expression of lipid metabolism genes	*Clostridium*, *Enterococcus*, *Coprobacillus*, *Lactococcus*, *Sutterella*, *Paraprevotella*, *Prevotella*, S24-7, *Treponema*, *Ruminococcus*	POP increased SCFA levels (isobutyric, butyric, and valeric acids) and regulated the expression of lipid metabolism-related genes (PPARα, ATGL, C/EBPα, C/EBPβ, FAS, SREBP-1c, FABP4). [88].
*Chrysanthemum morifolium* Ramat	C. morifolium extract	HFDF C57BL/6 J mice	Body weight, Liver TC, TG, FBG, ALT, AST, GTT, ITT, LDL-C	Bacteroidota, Actinobacteriota, Lachnospiraceae, Oscillospiraceae, Bifidobacteriaceae, Firmicutes, *Faecalibaculum*	C. morifolium extract promoted nuclear localization of PPARα and activated fatty acid oxidation (FAO) pathway. [109].
*Gardenia jasminoides Ellis*	GC	HFDF SD rats	Body weight, TC, TG, LDL-C, HDL-C, Hepatic oxidative stress indicators	ratio of Firmicutes/Bacteroidetes, *Lactobacillus*, *Akkermansia*, *Lactobacillus*, *Bacteroides*, Proteobacteria, Desulfobacterota, Rikenellaceae_RC9_gut_group	GC suppressed TLR4, Myd88, and NF-κB expression, improving intestinal barrier function and reducing inflammation. [110].
*Smilax china L*	JGTCs	HFD + STZ Wistar rats	FBG, PBG, OGTT, AUC, TC, TG, LDL-C, AST, ALT, Hepatic lipid accumulation, Liver histopathological inflammation, Inflammatory cytokines	*Aerococcus*, *Blautia*, *Coprococcus*, *Escherichia-Shigella*, *Prevotella*, Prevotellaceae_UCG-001, *Flavonifractor*	1. JGTCs activated FXR signaling, suppressed hepatic lipogenic genes (SREBP-1c, FAS), and improved bile acid metabolism 2. JGTCs suppressed the expression of inflammatory cytokines (IL-1β, TNF-α, IL-6). [111].

HFDF represents a high-fat, high-sugar diet; HFHS represents high-fat/high-sucrose-induced obese; HFD refers to a high-fat diet; STZ stands for *Streptozotocin*; *Sal75* *Salvia miltiorrhiza* 75% ethanol extract, *LJP61A* *Laminaria japonica* polysaccharide, *PLS* *Pueraria lobata* starch, *AOE* *Alisma orientale* 80% ethanol extract, *WESB* *Radix scutellariae* water extract, *MLEs* *Mulberry leaf* water extracts, *PCPs-I* *Polygonum cuspidatum* polysaccharides, *BBR* Berberine, *Agl* *Gypenoside* aglycones, *PLP* *Plantago asiatica L*. polysaccharide, *FFL* Fermented *Flos Lonicera*, *MDG-1* an *Ophiopogon* polysaccharide, *MFE95* *Mulberry* fruit 95% ethanol extract, *DH* *Dendrobium huoshanense*, *RHUB* *Rhubarb* root extract, rich in polyphenols, *CA* Chicoric acid, *AE* Polysaccharides from *Apocynum venetum* leaves, *BBBP* Biotransformed bear bile powder, *POP* *Polygonatum odoratum* polysaccharides, *C. morifolium extract* *Chrysanthemum morifolium* Ramat aqueous extract, *GC* *Gardenia jasminoides* crocin extract, *JGTCs* Jingangteng capsules

In addition, some herbal extracts can indirectly increase SCFA levels by promoting the proliferation of SCFA-producing gut microbiota. For example, *Salvia miltiorrhiza* 75% ethanol extract [89], *Alisma orientale* 80% ethanol extract (AOE) [90], *Polygonum cuspidatum* polysaccharide (PCP-I) [91], and *Rhubarb* root extract (RHUB, rich in polyphenols) [92] have been shown to significantly increase SCFA-producing bacteria. For example, PCPs-I increased the abundance of Bacteroides and *Lachnospiraceae*, both of which are known butyrate producers [91]. AOE was found to promote the growth of *Lactobacillus* and *Ruminococcus*, bacteria capable of synthesizing acetate, propionate, and butyrate [90]. Furthermore, Xie et al. [93] reported that *gypenoside aglycones* improved the gut microbiota composition and exerted multiple regulatory effects, including downregulation of lipid synthesis genes (FAS, PPARγ, HMGCS1, SREBP-1c), modulation of bile acid metabolism via activation of the FXR–FGF15 signaling pathway, and enhancement of antioxidant capacity through upregulation of superoxide dismutase activity (SOD) expression. These effects led to significant reductions in the serum TC, TG, and LDL-C cholesterol (LDL-C) levels and attenuated hepatic lipid accumulation in high-fat diet-fed SD rats.

As a representative cold/cool-property SHM, RHUB is known for its clear heat and eliminate stagnation, promote bowel movements, and circulation improvement properties [94]. Marion et al. [92] reported that RHUB modulated gut dysbiosis induced by a high-fat, high-sugar diet in C57BL/6 J male mice by increasing the abundance of *Akkermansia muciniphila*, *Parabacteroides*, and *Erysipelatoclostridium* while decreasing *Ruminococcus* and *Peptococcus*. These microbial changes were accompanied by anti-inflammatory effects (reduced expression of RANTES, TNF-α, IL-6, and IFN-γ), upregulation of antimicrobial peptide genes (Reg3γ and Pla2g2), and increased intestinal barrier integrity. Additionally, RHUB upregulated *Ucp1* expression, thereby promoting energy metabolism. *Dendrobium huoshanense*, a dual-purpose medicinal and edible SHM that not only clears heat but also replenishes the body’s fluid loss associated with heat syndromes, was shown by Ma et al. [95] to promote the proliferation of beneficial bacteria (*B. pseudolongum*, *B. animalis*, and *Allobaculum*) and reduce the number of harmful bacteria (*Helicobacter hepaticus*, *Rothia mucilaginosa*, and *Clostridium celatum*). It also facilitates the excretion of deoxycholic acid and regulates amino acid metabolism, significantly reducing serum TG and TC levels in mice fed a high-fat, high-sugar diet.

### Warm/heat-property SHM

Warm/heat-property SHMs are commonly used to dispel pathogenic cold, thus alleviating cold-related syndromes. Our study indicates that many warm/heat-property SHMs exert lipid-regulating effects by increasing the expression of intestinal tight junction proteins, preserving gut barrier integrity, and reducing the serum levels of LPS (Table 2). Representative SHMs include *hydroxysafflor yellow A* [104], *Akebia saponin D* [105], aqueous extract of fermented *Eucommia ulmoides* leaves (FELE) [106], *ginsenoside* extract [107], *Panax notoginseng* saponins [108], *hawthorn* 95% ethanol extract [109], *curcumin* [110], water extract of *Caulis spatholobi* [111], *Myristica fragrans* extract [112], *red yeast* rice aqueous extract [113], ethanol extract of *Atractylodis macrocephalae Rhizoma* [114], and volatile oil of *Amomum villosum* [115].

**Table 2:** Summary of studies on warm/heat-property SHMs regulating lipid metabolism via the gut microbiota

SHM	Extract	Experimental subject	Improvement index	Changes in gut microbiota composition	Mechanisms
*Carthamus tinctorius L*	HSYA	HFDF C57BL/6 J mice	Body weight, Epididymal fat weight and fat area, Hepatic lipid droplet size, Fecal fat content, FBG, HOMA-IR, Expression of inflammatory cytokines	ratio of Firmicutes/Bacteroidetes, Verrucomicrobia, *Akkermansia*, *Romboutsia*, *Butyricimonas*, *Alloprevotella*, Lachnospiraceae, *Lactobacillus*	1. HSYA increased the levels of SCFAs in feces 2. HSYA enhanced intestinal barrier integrity by increasing the expression of colonic tight junction protein ZO-1 3. HSYA suppressed the expression of pro-inflammatory cytokines (TNF-α, IL-1β, IL-6) in the liver. [112].
*Dipsacus asper*	ASD	HFDF C57BL/6 J mice	Body weight, Fasting Insulin, TG, LDL-C, FBG, HOMA-IR, NEFA, Hepatic oxidative stress indicators	*Alistipes*, *Prevotella*, *Butyricimonas*, Ruminococcaceae, *Bifidobacterium*	1. ASD upregulated the expression of tight junction proteins and improved intestinal barrier integrity 2. ASD downregulated the PPAR-γ/FABP4 pathway, thereby reducing intestinal lipid absorption 3. ASD increased the abundance of butyrate-producing bacteria. [113].
*Schisandra chinensis* fruit	SCF	Obese adult women	Body weight,Fat mass percentage, WC, BMI, FBG, TC, TG, HDL-C, AST, ALT	*Akkermansia*, *Roseburia*, *Prevotella*, *Bifidobacterium*, *Ruminococcus*	SCF treatment significantly enriched SCFAs producing bacteria. [135].
*Eucommia ulmoides* leaves	FELE	HFD Wistar rats	Body Weight, Liver Index, Adipose Index, TC, TG, LDL-C, HDL-C, ALT, Hepatic oxidative stress indicator, Expression of inflammatory cytokines	ratio of Firmicutes/Bacteroidetes, *Lactobacillus*, *Romboutsia*, *Bacteroides*, *Roseburia*, *Ruminococcus*, *Lachnospira*	1. FELE regulated BAs metabolism 2. FELE reduced inflammation and oxidative stress 3. FELE modulated lipid metabolism-related gene expression. [114]
Linderae Radix	LREE	HFDF SD rats	TC, TG, LDL-C, HDL-C, ALT, AST, Primary/secondary bile acid metabolism	ratio of Firmicutes/Bacteroidetes, Actinobacteria	LREE improved lipid levels via modulation of BA metabolism. [130].
*Panax ginseng*	GE	HFDF C57BL/6 J mice	Body weight, TC, TG, LDL-C, Liver TC, TG, AST, ALT, Hepatic lipid metabolism-related genes, Expression of inflammatory cytokines	ratio of Firmicutes/Bacteroidetes, Muribaculaceae, *Parabacteroides*, *Akkermansia*, *Ruminococcus torques* group, Lachnospiraceae, *Helicobacter*	1. GE treatment significantly enriched SCFAs producing bacteria 2. GE upregulated tight junction proteins (ZO-1, occludin) and reduced circulating LPS levels, thus enhancing intestinal barrier integrity 3. GE exhibited anti-inflammatory activity by suppressing NF-κB pathway and cytokine production. [115].
*Corydalis yanhusuo* W.T. Wang	THP	ApoE −/− mice	TC, TG, HDL-C, LDL-C, Atherosclerotic plaque area, Hepatic cholesterol content, Expression of inflammatory cytokines	Verrucomicrobiaceae, Clostridia, Verrucomicrobiae, Verrucomicrobiales, Clostridi	1. THPincreased the levels of SCFAs in feces 2. THP regulate bile acid metabolism through modulation of intestinal microbiota and FXR signaling pathways. [132].
*Panax notoginseng* (Burk.) F.H. Chen	PNS	HFDF C57BL/6 J mice; *ob/ob* mice	Body weight, FBG, TG, TC, HDL-C, LDL-C, FFA, AST, ALT, Expression of inflammatory cytokines, Liver histopathology	ratio of Firmicutes/Bacteroidetes, *Parabacteroides distasonis*	1. PNS increased the levels of SCFAs in feces 2. PNS increased the expression of tight junction proteins (ZO-1 and Claudin-1). [116].
*Crataegus pinnatifida* (hawthorn fruit)	HEE	HFDF C57BL/6 J mice; PG mice	Body weight, FBG, ALT, AST, ALP, TG, TC, LDL-c, HDL-c, Expression of inflammatory cytokines, Hepatic lipid metabolism-related genes	ratio of Firmicutes/Bacteroidetes, *Lactobacillus*, Lachnospiraceae, *Ruminiclostridium*, *Streptococcus*, *Stenotrophomonas*, *Enterobacter*	1. HEE upregulated tight junction proteins (ZO-1, occludin) and reduced circulating LPS levels, thus enhancing intestinal barrier integrity 2. HEE increased the levels of SCFAs in feces 3. HEE improved lipid levels via modulation of BA metabolism. [117].
Dried *Citrus* Peel (Chenpi)	CP	C57BL/6 J mice	Body weight, Liver-to-body weight ratio, TG, TC, LDL-c, HDL-c, oxidative stress indicator, Abdominal adipose tissue to body weight ratio, Subcutaneous adipose tissue to body weight ratio, Perirenal adipose tissue to body weight ratio	ratio of Firmicutes/Bacteroidetes, Muribaculaceae, *Muribaculum*	CP increased the levels of SCFAs in feces. [136].
*Curcuma longa L*	Cur	HFDF C57BL/6 J mice	Body weight, Food intake, Energy intake, Liver index, Subcutaneous/epididymal fat pad mass, HOMA-IR, GTT, ITT, TG, TC, ALT, AST, liver TG, TC, Hepatic lipid metabolism-related genes, FSI	ratio of Firmicutes/Bacteroidetes, *Bacteroides*, *Parabacteroides*, *Alistipes*, *Alloprevotella*, *Akkermansia*, *Desulfovibrio*, Ruminococcaceae_UCG-014, *Lactobacillus*	1. Cur increased the levels of SCFAs in feces 2. Cur ameliorated endotoxemia by lowering serum LPS levels. [118].
*Radix Astragali*	AS-IV	HFSD Kunming mice	Body weight, Food intake, Water intake, FBG, FIN, HOMA-IR, OGTT, AUC, TG, LDL-C, HDL-C, oxidative stress indicator, Histopathological scoring (liver and pancreas)	ratio of Firmicutes/Bacteroidetes, *Alistipes*, *Odoribacter*, *Lactobacillus*, *Bacteroides*, *Oscillibacter*	Enhanced SCFA levels and stimulated insulin-related signaling pathways (AMPK/SIRT1, PI3K/AKT). [137].
Caulis Spatholobi	WECS	HFDF C57BL/6 J mice	Body weight/Body fat ratio, Visceral fat mass, GTT, ITT, TC, TG, LDL-C, HDL-C, Lipogenesis gene, Hepatic inflammatory cytokines, Rectal temperature, Brown adipose markers	ratio of Firmicutes/Bacteroidetes, Proteobacteria, *Bifidobacterium*, *Lactobacillus*, *Parabacteroides*, *Anaerotruncus*	1. WECS suppressed lipogenesis and promoted lipid catabolism via MAPK/AMPK activation 2. WECS suppressed the expression of inflammatory cytokines (TNF-α, IL-6, IL-1β). [119].
*Perilla frutescens* (L.) Britt	PO	Diabetic KKAy mice	Body weight, Liver weight, Perirenal fat weight, Epididymal fat weight, FBG, FIN, TC, TG, LDL-C, HDL-C, Liver histopathology	*Blautia*, *Lactobacillus*, *Akkermansia*	PO is proposed to enhance the production of secondary BAs and SCFAs via bile–microbiota interactions. [133].
Fenugreek	WFS	HFDF C57BL/6 J mice	Body weight, Body fat, 40 min OGTT, FBG, LDL-c, HDL-c, TC	Clostridium cluster XIVa, Cluster IV, Runinococcacea	1. WFS increaseof SCFAs in feces 2. WFS modulates the metabolism and fecal excretion of secondary BAs. [134].
*Myristica fragrans* Houtt	MFE	HFDF C57BL/6/J mice	LDL-c, TC, TG, AST, ALT, FBG, Hepatic lipid accumulation, inflammatory cytokines	*Akkermansia*, *Blautia*, *Bifidobacterium*, *Adlercreutzia*, SMB53, *Allobaculum*, *Odoribacter*	1. MFE reduced hepatic expression of pro-inflammatory cytokines (TNF-α, IL-6, IL-1β) 2. MFE increased intestinal tryptophan metabolites and activated hepatic AhR to regulate lipid metabolism. [120].
Red yeast rice	RYR	HFDF Apoe⁻/⁻ mice	Atherosclerotic lesion area, LDL-c, TC, TG, Intestinal villus length	Bacteroidetes, *Bacteroides*, *Anaeroplasma*, *Alistipes*, *Flavonifractor*	1. RYR increased the expression of intestinal tight junction proteins (JAM-1 and occludin), thereby enhancing intestinal barrier integrity 2. RYR increased the abundance of SCFA-producing gut bacteria, particularly Bacteroides 3. RYR suppressed the expression of pro-inflammatory cytokines, including TNF-α and IL-1β. [121].
*Atractylodes macrocephala* Koidz*.*, rhizome	AMK	*db/db* mice	Body Weight, FBG, HOMA-IR, TC, TG, Inflammatory cytokines	*Bacteroides thetaiotaomicron*, *Methanobrevibacter smithii*, *B. thetaiotaomicron*, *M. smithii*	1. AMK suppressed inflammatory cytokine expression and reduced serum LPS levels 2. AMK activated the GLP-1R/PI3K/PDX-1 signaling pathway. [122].
*Rosa roxburghii Tratt*	FBB	HFDF + STZ Kunming mice	Body weight, FBG, GSP, Insulin level, HOMA-IR, TG, TC, LDL-c, HDL-c, Scr, UA	*Dobacterium*, *Prevotella*, *Ruminococcus*, *Clostridium*, *Allobaculum*, *Adlercreutzia*, *Helicobacter*	FBB increaseof SCFAs in feces. [138].
*Amomum villosum* Lour	VOAV	HFDF SD rats	Body weight, LDL-c, TC, TG, HDL-c, FFA, AST, ALT, Liver Index, Inflammatory cytokines	ratio of Firmicutes/Bacteroidetes, *Lactobacillus*, *Prevotella*, *Clostridium*, *Faecalibacterium*, *Oscillospira*	1.VOAV suppressed pro-inflammatory cytokines (TNF-α, IL-6, IL-1α) and increased anti-inflammatory IL-10 2. Inhibited pro-inflammatory cytokines and enhanced intestinal tight junction proteins to reduce LPS leakage. [137].

HFDF and HFSD denote high-fat/high-sugar diets, while STZ refers to *Streptozotocin*; *HSYA* Hydroxysafflor yellow A, *ASD* Akebia saponin D, *SCF* Aqueous extract of *Schisandra chinensis* fruit, *FELE* Aqueous extract of fermented *Eucommia ulmoides* leaves, *LREE* *Linderae Radix* 70% ethanol extract, *GE* *Ginsenoside* extract, *THP* Tetrahydropalmatine, *PNS* *Panax notoginseng* saponins, *HEE* *Hawthorn* 95%Ethanol Extract, *CP* *Chenpi* powder, *Cur* Curcumin, *AS-IV* Astragaloside IV, *WECS* Water Extract of *Caulis Spatholobi*, *PO* *Perilla* oil, *WFS* Whole *fenugreek* seed, *MFE* *Myristica fragrans* extract, *RYR* Red yeast rice, *AMK* Ethanol extract of *Atractylodes macrocephala* Koidz., rhizome, *FBB* *Rosa roxburghii*-edible fungi fermentation broth B, *VOAV* Volatile oil of *Amomum villosum*

Xu et al. [108] reported that *Panax notoginseng* saponins improve gut barrier function by modulating the gut microbiota, inhibiting the TLR4/AMPKα signaling pathway, and increasing tight junction protein expression, resulting in significantly reduced serum levels of TG, TC, and LDL-C. Another study revealed that the *hawthorn* 95% ethanol extract significantly improved the gut microbiota composition, reduced blood glucose and lipid levels, and alleviated hepatic histopathological damage induced by a high-fat diet. These effects are potentially associated with suppressed inflammatory cytokine release, decreased serum LPS levels, increased SCFA production, and the regulation of bile acid metabolism [109]. Further research revealed that *hawthorn* polysaccharides significantly suppressed weight gain and improved liver function in a NAFLD model, possibly through gut microbiota modulation and increased SCFA levels [116].

Red yeast rice extract, another typical warm/heat-property HM, has shown promising lipid-lowering effects. Xuezhikang, a TCM preparation made from red yeast rice extract, has demonstrated lipid-regulating, anti-inflammatory, antioxidant, endothelium-protective, antiplatelet, anti-NAFLD, insulin-sensitizing, and renoprotective effects in both animal and clinical studies [117]. Dong et al. reported that red yeast rice aqueous extract modulated the gut microbiota in high-fat, high-fructose diet-fed Apoe −/− mice by increasing *Bacteroidetes*, *Bacteroides*, *Anaeroplasma*, *Alistipes*, and *Flavonifractor* while reducing serum total cholesterol (TC) and low-density lipoprotein cholesterol (LDL-C) levels and atherosclerotic plaque areas [113]. A representative warm/heat-property HM, *Lindera aggregata* exhibits kidney-warming, cold-dispersing, and qi-regulating analgesic effects, and is considered effective in alleviating cold-related syndromes. Jiang et al. [118] reported that the ethanol extract of *Lindera aggregata* lowers the aberrantly elevated abundances of Firmicutes and Actinobacteria while increasing Bacteroidetes, thereby reshaping the bile salt hydrolase-mediated bile acid profile, restoring bile acid reabsorption pathways, and upregulating hepatic CYP7A1. These effects collectively accelerate cholesterol elimination and confer lipid-lowering and hepatoprotective benefits. Another commonly used warm/heat-property HM, *Panax ginseng*, primarily functions to tonify Qi, manifested by enhancing systemic energy, nourishing the blood, calming emotions, and improving cognitive performance [119]. Liang et al. [107] demonstrated that ginsenosides enriched *Muribaculaceae*, *Parabacteroides*, *Akkermansia*, and *Ruminococcus_torques_group* while decreasing *Lachnospiraceae* and *Helicobacter*, thereby suppressing the NF-κB/IκB pathway and regulating lipid metabolism via the AMPK pathway (CPT-1a, SREBP-1c) in high-fat diet-fed mice.

Other warm/heat-property SHMs, such as the tetrahydropalmatine from *Corydalis yanhusuo* [120], *Perilla* seed oil [121], *Trigonella foenum-graecum* powder [122], and FELE [106], also exert lipid-lowering effects by modulating the gut microbiota and increasing secondary bile acid levels. Notably, FELE not only regulates bile acid metabolism via activation of the FXR/LXRα/CYP7A1 pathway but also exerts anti-inflammatory effects (reducing NF-α, IL-6, and MCP-1), alleviates oxidative stress (modulating MDA, SOD, and CAT), and regulates key lipid metabolism genes (PPARα, CPT1A, LXRα, CYP7A1, and SREBP-1c) [106].

### Neutral-property SHMs

Neutral-property SHMs are characterized by their balanced properties, neither cold nor hot, and are considered effective in supporting normal physiological functions. In our research on neutral-property SHMs, we found they exert notable lipid-lowering effects through multiple pathways by modulating the gut microbiota (Table 3).

**Table 3:** Summary of studies on neutral-property SHMs improving lipid metabolism via gut microbiota

SHM	Extract	Experimental subject	Improvement index	Changes in gut microbiota composition	Mechanisms
*Poria cocos*	PCO	HFDF C57BL/6/J mice	FBG, HOMA-IR, FIN, Adipocyte size, Visceral fat weight, HOMA-IS, Expression of lipid metabolism-related genes	ratio of Firmicutes/Bacteroidetes, Ruminococcaceae, Anaeroplasmataceae, Lactobacillaceae, Rikenellaceae	PCO suppressed pro-inflammatory cytokine expression and enhanced tight junction protein levels (ZO-1, Occludin, Claudin-1) 2. PCO modulated intestinal SCFAs, BAs, and tryptophan metabolites 3. PCO suppressed fatty acid synthesis-related genes, including FASN and DGAT. [150].
*Polygonatum kingianum* Coll. et Hemsl	ESP	HFDF C57BL/6/J mice	Body weight, Energy intake, Lee’s index, Liver index, iWAT index, TG, TC, NEFA, LDL-C, NEFAs, FBG, FIN, Inflammatory cytokines, Hepatic lipogenesis-related genes	ratio of Firmicutes/Bacteroidetes, *Dubosiella*, *Desulfovibrio*, *Acinetobacter*, *Akkermansia*, *Bifidobacterium*, *Lactobacillus*	1. ESPincreaseof SCFAs in feces 2. ESP suppressed pro-inflammatory cytokines (IL-6, TNF-α, IL-1β) in obese mice. [155].
*Ganoderma lucidum*	GLP	HFDF + STZ Kunming mice	Body weight, FBG, OGTT, HOMA-IR, TC, TG, LDL-C, HDL-C, Inflammatory cytokines, oxidative stress indicator	*Aerococcus, Proteus, Ruminococcus, Corynebacterium, Blautia, Parabacteroides, Bacteroides, Dehalobacterium*	1. GLP increased fecal butyric acid levels in T2DM rats 2. GLPsuppressed inflammatory cytokines (IL-1β, IL-6, CRP) 3. GLP enhanced antioxidant enzyme activity and reduced oxidative stress. [156].
Lotus leaf	Nuciferine	HFDF Kunming mice	Body weight, Lee's index, Food intake, FBG, Inflammatory cytokines, Hepatic lipid metabolism-related gene expression, Insulin signaling proteins, ALP, ALT, AST, TG, TC, HDL-C, LDL-C	ratio of Firmicutes/Bacteroidetes, *Lactobacillus*, Prevotellaceae_UCG-001, *Desulfovibrio*, *Mucispirillum*, Proteobacteria	1. Nuciferine activated the AMPK signaling pathway 2. Nuciferine suppressed inflammation, increased tight junction proteins, and reduced serum LPS levels. [143].
*Rosa laevigata* Michx	RLPs	HFDF SD rats	Body weight, Abdominal circumference, Lee's index, FBG, TC, TG, TC, TG, HDL-c, LDL-c, FFA, ALT, AST, GGT, FBG, OGTT, HOMA-IR, Genes related to hepatic lipid metabolism, Inflammatory cytokines, oxidative stress indicator	ratio of Firmicutes/Bacteroidetes, *Bacteroides*, *Alistipes*, *Prevotella*, *Akkermansia*, *Butyricimonas*	RLPs increaseof SCFAs in feces RLPs regulated the expression of hepatic lipid metabolism-related genes (e.g., FADS2, Scd-1, Acox3) 3. RLPs suppressed the expression of pro-inflammatory cytokines (TNF-α, IL-1β, IL-6). [157].
*Vaccinium vitis-idaea*	LB	HFDF Apoe-/- mice	TC, TG, LDL-VLDL, Body weight, Atherosclerotic plaque area, Hepatic bile acid synthesis gene, Inflammatory cytokines, HOMA-IR	*Bacteroides, Parabacteroides, Clostridium, Akkermansia muciniphila, Mucispirillum, Oscillospira, Bilophila, Turicibacter, Lactococcus*	1. LB increased bile acid synthesis in the liver by upregulating Cyp7a1 gene expression 2. LB increased the production of propionic acid in the cecum. [96].
*Ginkgo biloba*	GB	HFDF ApoE −/− mice	Atherosclerotic plaque area, TC, TG, VLDL-C, LDL-C, HDL-C, FBG, Inflammatory cytokines, Liver TG, TC	Bacteroidetes, *Bacteroides*, *Helicobacter*, *Roseburia*, Deferribacteres	1. GB reduced TMAO production by inhibiting FMO3 expression 2. GB suppressed pro-inflammatory cytokines, including hs-CRP, IL-6, and TNF-α. [154].
*Grifola frondosa*	GF95	HFDF Wistar rats	Body weight, Hepatic lipogenesis genes, TC, TG, LDL-C, HDL-C, AST, FFA, Liver histopathological changes	*Intestinimonas, Butyricimonas*	GF95 promoted bile acid secretion 2. GF95 increased SCFA production and activated AMPK signaling 3. GF95 enhanced antioxidant capacity(SOD, GSH-Px). [125].
*Lycium barbarum*	LBP	Kunming mice	Body weight, TC, TG, LDL-C, HDL-C, Inflammatory cytokines, oxidative stress indicator	*Lactobacillus, Lactococcus, Ruminococcus, Mucispirillum, Allobaculum*	LBP promoted SCFA productianced fatty acid oxidation. [158].
*Glycyrrhiza uralensis*	GUP	HFDF + STZ C57BL/6/J mice	Body weight, FBG, OGTT, HOMA-IR, G, TC, LDL-C, HDL-C, FFA, AST, ALT, UREA, Scr, Inflammatory cytokines, oxidative stress indicator	*Akkermansia*, *Lactobacillus*, *Romboutsia*, *Faecalibaculum*, Lachnospiraceae_NK4A136_group, *Bacteroides*, *Escherichia–Shigella*, *Clostridium* sensu stricto 1	1. GUPincreaseof SCFAs in feces 2. GUP suppresses the expression of inflammatory cytokines, reduces serum LPS levels. [159].
*Hirsutella sinensis*	HSM	HFDF C57BL/6/J mice	Body weight,Visceral fat pad weight, HOMA-IR, FBG, TG, Inflammatory cytokines	*Parabacteroides goldsteinii, Escherichia coli, Shewanella algae, Clostridium cocleatum, Anaerotruncus colihominis*	1. HSM suppresses the expression of pro-inflammatory cytokines (IL-1β, TNF-α) 2. HSM increases tight junction protein (ZO-1) expression and reduces serum LPS concentration 3.HSM regulates the expression of lipid metabolism-related genes in adipose and hepatic tissues. [97].
*Dioscorea* spp.	Dioscin	HFDF SD rats	Body weight, TG, TC, Liver histopathological changes	*Globicatella*, *Phaseolaretobacterium*, *Pseudochrobacterium*, Prevotellaceae	Dioscin regulates BAs and tryptophan metabolism. [98].
*Smilax china* L	SCP	HFDF C57BL/6/J mice	Body weight, Liver and adipose tissue weights, TG, TC, LDL-C, HDL-C, Liver lipid accumulation, Hepatic lipogenesis genes	ratio of Firmicutes/Bacteroidetes, Proteobacteria, Actinobacteria, *Faecalibaculum*, *Allobaculum*, Coriobacteriaceae UCG-002, Lachnospiraceae NK4A136_group, Ruminococcaceae UCG-014, Prevotellaceae UCG-004	1. SCP reduced pro-inflammatory and pathogenic bacteria 2. SCP promotes SCFA-producing bacteria and regulates lipid metabolism via AMPK/Sirt1/PPARα. [99].

HFDF and STZ are defined as in Tables 1 and 2; *PCO* *Poria cocos* oligosaccharides, *ESP* Enzymatic saccharifying extracts of *Polygonatum kingianum*, *GLP* *Ganoderma lucidum* polysaccharide, *RLPs* Low molecular weight polysaccharides from *Rosa laevigata* Michx, *LB* Lingonberry, *GB* Ginkgolide B, *GF95* 95% ethanol extract of *Grifola frondose*, *LBP* *Lycium barbarum* polysaccharide, *GUP* *Glycyrrhiza uralensis* polysaccharide, *HSM* *Hirsutella sinensis* mycelium, *SCP* *Smilax china* L*.* polysaccharide

For example, lotus leaf, derived from the dried leaves of *Nelumbo nucifera*, is widely used to treat metabolic disorders such as hyperlipidemia, diabetes, obesity, and fatty liver disease [127, 128]. It contains various bioactive compounds, including alkaloids, flavonoids, terpenoids, and steroids [129, 130]. According to Zhu et al. [131], nuciferine (a major alkaloid) can regulate the gut microbiota and exert anti-inflammatory effects via the TLR4/MyD88/NF-κB pathway while enhancing intestinal barrier function by upregulating tight junction proteins (*claudin*, *ZO-1*, and *occludin*). Additionally, nuciferine activates the AMPK pathway, suppresses hepatic lipogenesis-related genes (SREBP-1c, FAS), and promotes lipolytic gene expression (ATGL, HSL). In high-fat diet-induced Kunming mice, nuciferine significantly reduced the serum levels of ALP, ALT, AST, TG, TC, and LDL-C; lowered blood glucose; and mitigated visceral fat accumulation and weight gain.

*Poria cocos*, the dried sclerotium of *Poria* (family Polyporaceae), is traditionally used to increase kidney fluid metabolism, restore internal fluid balance, improve spleen and stomach function, and calm the mind [132–134]. Zhu et al. [135] reported that *Poria cocos* oligosaccharides modulated the gut microbiota by decreasing the Firmicutes/Bacteroidetes (F/B) ratio and reducing *Ruminococcaceae* and *Anaeroplasmataceae* while increasing the abundance of *Lactobacillus* and *Clostridium*. This intervention improved blood glucose levels and reduced body weight and visceral fat. The underlying mechanisms may involve the inhibition of proinflammatory cytokines (TNF-α and IL-1β), a reduction in LPS leakage, and the regulation of SCFA (e.g., butyrate), bile acid, and 5-hydroxytryptamine metabolism.

*Ginkgo biloba* leaf, one of the oldest known medicinal plants, contains flavonoids and terpene lactones as its main bioactive compounds [136]. It has been widely used to increase blood circulation, support microcirculation, improve lung function, and regulate lipid metabolism [137, 138]. Studies have shown that *Ginkgo biloba* extract can reshape the gut microbial composition, suppress FMO3 expression, and thereby influence the TMA/TMAO metabolic pathway to reduce TMAO production. This mechanism is closely associated with reductions in serum TC, TG, very LDL-C (VLDL-C), LDL-C, hsCRP, and fasting blood glucose, along with an increase in HDL-C and reduced formation of atherosclerotic plaques[139].

Several other neutral-property herbal extracts have also demonstrated lipid-lowering effects through the modulation of the gut microbiota. These include the enzymatically hydrolyzed *Polygonatum sibiricum* extract [140], *Ganoderma lucidum* polysaccharides [141], low-molecular-weight polysaccharides from *Rosa laevigata* [142], *Lycium barbarum* polysaccharide [143], and *Glycyrrhiza uralensis* polysaccharide [144]. These compounds have been shown to increase fecal SCFA levels and suppress inflammatory cytokines to varying degrees. In addition, *Lingonberry* [145] not only increases propionate production but also upregulates *Cyp7a1* expression, thereby promoting bile acid synthesis. Polysaccharides from *Hirsutella sinensis* mycelium significantly reduce serum LPS levels and regulate key genes involved in lipid synthesis (*SREBP-1c*, *FAS*, and *Acsl3*) in adipose and hepatic tissues, contributing to improved lipid metabolism [146].

## Conclusion and perspectives

As the “second genome” of the human body, the gut microbiota plays a vital role in maintaining host metabolic homeostasis, regulating immune responses, and preserving intestinal barrier integrity [14, 60, 150, 151]. In recent years, with the increasing prevalence of dyslipidemia and its associated risk of cardiovascular diseases [152, 153], increasing attention has been given to the use of SHMs to modulate the gut microbiota and its metabolites as a strategy to lower blood lipid levels. This study systematically elucidates the mechanisms by which SHMs regulate lipid metabolism through the modulation of the gut microbiota and its key metabolites—SCFAs, LPS, BAs, and TMAO (Fig. 1). Among them, the SCFA and LPS pathways play pivotal roles in lipid regulation, anti-inflammatory responses, and maintenance of intestinal barrier integrity, representing core mechanisms through which SHMs exert lipid-lowering effects. Furthermore, on the basis of the TCM theory of the “Four qi” (cold, hot, warmth, and coolness), this study categorized SHMs according to their cold/hot properties and clinical indications, providing a theoretical foundation for individualized treatment strategies. These findings emphasize the importance of selecting targeted herbal therapies on the basis of patients’ specific pathological patterns to optimize both efficacy and safety in clinical practice.

This study provides a comprehensive review of 57 SHMs with different cold/hot properties that regulate the gut microbiota to improve lipid metabolism, including 24 cold/cool-property, 20 warm/heat-property, and 13 neutral-property herbs. Our analysis revealed that a decrease in the Firmicutes-to-Bacteroidetes (F/B) ratio, along with an increased abundance of *Akkermansia*, *Lactobacillus*, *Ruminococcaceae*, and *Clostridium*, was a common microbiota-related feature shared across all lipid-lowering SHMs, indicating a universal gut microbiota-modulating effect. However, distinct microbial modulation patterns were observed among the different properties of SHMs. Cold/cool-property SHMs predominantly affect taxa such as *Psychrobacter* and *Quinella*, which are associated with environmental stress tolerance, cold adaptation, and specific metabolic pathways, including amino acid metabolism and hydrogen sulfide production [154, 155]. These findings suggest that cold/cool-property SHMs may exert lipid-lowering effects by modulating the metabolic activity of microbiota adapted to environmental stress. Warm/heat-property SHMs uniquely enrich *Akkermansia muciniphila* and SCFA-producing bacteria such as *Romboutsia* and *Butyricimonas*, implying that their regulatory effects on host metabolism may involve the enhancement of gut barrier function, the modulation of immune responses, and the stimulation of microbial fermentation [156, 157]. In contrast, neutral-property SHMs have a broader regulatory influence on various bacterial taxa, with particularly notable effects on *Proteus* and *Lactobacillus*, suggesting that these SHMs may play a more integrative role in maintaining gut microbial homeostasis [158, 159].

In addition, unlike Western lipid-lowering drugs, which typically act on single targets, HMs display multipathway, multitarget, and multilevel mechanisms in lipid metabolism regulation. For example, cold/cool-property SHMs such as *Laminaria japonica*, *Scutellaria baicalensis*, *Polygonum cuspidatum*, *Plantago asiatica*, *Andrographis paniculata*, and *Rheum palmatum* not only reduce blood lipid levels but also modulate glucose‒lipid metabolism, showing additional benefits such as hypoglycemic effects and improved insulin sensitivity [83, 86, 91, 92, 96, 97]. Warm/heat-property SHMs—including *Carthamus tinctorius*, *Dipsacus asper*, *Panax ginseng*, *Panax notoginseng*, *Crataegus pinnatifida*, *Curcuma longa*, *Astragalus membranaceus*, and *Caulis Spatholobi*—exhibit lipid-lowering effects as well as reductions in body weight, visceral fat accumulation, and hepatic histopathological alterations [104, 105, 107–111, 125]. Neutral-property SHMs such as *Poria cocos*, *Rosa laevigata*, *Nelumbo nucifera*, and *Ganoderma lucidum* primarily exhibit anti-inflammatory and antioxidant properties and regulate the expression of lipid metabolism-related genes, highlighting their therapeutic value in addressing metabolic disorders [131, 135, 141, 142].

Although SHM has demonstrated promising potential in regulating lipid metabolism via modulation of the gut microbiota, aligning the cold/hot properties of SHM with those of the human body remains a significant challenge—especially for nonspecialist practitioners or the general public—according to TCM fundamentals. Therefore, future research should focus on scientifically refining and integrating the TCM theory of cold/hot with modern medical frameworks, with the aim of developing more precise, accessible, and operational personalized lipid-lowering strategies. In particular, it is essential to improve the tools and methods used to identify the cold–heat syndrome in individuals with hyperlipidemia. Such methods should be user-friendly, accurate, and readily applicable in practice, enabling even non-professionals to select appropriate TCM interventions.

Moreover, future research should prioritize the construction of a comprehensive HM–gut microbiota–lipid metabolism database. This requires the systematic collection and analysis of clinical efficacy data on the ability of SHM to modulate the gut microbiota and treat hyperlipidemia while accounting for interindividual differences, regional diversity, and dietary habits to ensure broad applicability. These high-quality clinical datasets will serve as a valuable foundation for training artificial intelligence-based models for personalized HM prescriptions, potentially accelerating the modernization and global application of TCM. This goal will demand cross-regional and interdisciplinary collaboration involving fields such as TCM, microbiology, metabolomics, and data science to expand both the depth and breadth of future research.

In conclusion, compared with multiherb formulations, SHMs—due to their traceable active components and mechanisms of action—are more easily accepted and disseminated in regions where TCM is less developed. Therefore, their value in the prevention and treatment of hyperlipidemia and other metabolic disorders deserves greater attention and should be prioritized in future research and clinical practice.

## Data Availability

Not applicable.

## Abbreviations

TC: Total cholesterol
CYP7A1: Cholesterol 7α-Hydroxylase
TG: Triglycerides
UCP1: Uncoupling protein 1
HDL-C: High-density lipoprotein cholesterol
cAMP: Cyclic AMP
NAFLD: Nonalcoholic fatty liver disease
PKA: Protein kinase A
HM: Herbal medicine
FMO3: Flavin-containing monooxygenase 3
SHM: Single HM
TMA: Trimethylamine
TCM: Traditional Chinese medicine
MAPK: Mitogen-activated protein kinase
SCFA: Short-chain fatty acid
JNK: C-Jun N-terminal kinase
BA: Bile acid
FFAs: Free fatty acids
TMAO: Trimethylamine-N-oxide
LDL: Low-density lipoprotein
LPS: Lipopolysaccharide
ox-LDL: Oxidized LDL
AMPK: AMP-activated protein kinase
LDL-C: LDL cholesterol
HSL: Hormone-sensitive lipase
TLR4: Toll-like receptor 4
ATGL: Adipose triglyceride lipase
MLEs: *Mulberry leaf* water extracts
PPAR: Peroxisome proliferator-activated receptor alpha
MDG-1: *Ophiopogon* polysaccharide
SREBP: Sterol regulatory element-binding protein
PCPs-I: *Polygonum cuspidatum* polysaccharides
GLP-1: Glucagon-like peptide-1
RHUB: *Rhubarb* root extract
FXR: Farnesoid X receptor
SOD: Superoxide dismutase activity
TGR: Takeda G protein-coupled receptor 5
FELE: Aqueous extract of fermented *Eucommia ulmoides* leaves
FASN: Fatty acid synthase
VLDL-C: Very LDL-C
